# Can european law keep pace with biomedical innovation? The reinvention of transplantation regulation

**DOI:** 10.3389/ti.2026.16976

**Published:** 2026-06-16

**Authors:** Olivier Thaunat, Frank J. M. F. Dor, Umberto Cillo, Gabriel C. Oniscu, Ekaterine Berishvili

**Affiliations:** 1 Department of Transplantation, Nephrology and Clinical Immunology, Hospices Civils de Lyon, Edouard Herriot Hospital, Lyon, France; 2 Lyon-Est Medical Faculty, Claude Bernard University (Lyon 1), Lyon, France; 3 CIRI, INSERM U1111, Université Claude Bernard Lyon I, CNRS UMR5308, Ecole Normale Supérieure de Lyon, University Lyon, Lyon, France; 4 Erasmus MC Transplant Institute, Division of HPB and Transplant Surgery, Erasmus MC University Medical Center, Rotterdam, Netherlands; 5 Hepato-Pancreato-Biliary and Liver Transplant Surgery Unit, Padua University Hospital, Padua, Italy; 6 Department of Transplantation Surgery, Tema ARM, Karolinska University Hospital, Stockholm, Sweden; 7 Tissue Engineering and Organ Regeneration Laboratory, Department of Surgery, Faculty of Medicine, University of Geneva, Geneva, Switzerland; 8 Cell Isolation and Transplantation Centre, Department of Surgery, Geneva University Hospitals, Geneva, Switzerland

**Keywords:** biomedical innovation, European Union, ex-situ organ perfusion, transplantation regulation, xenotransplantation

Europe is rewriting the rules for a generation of therapies that did not exist when the current rules were drafted. Directive 2001/18/EC [1] on genetically modified organisms and Directive 2010/53/EU [2] on human organs were written for a biomedical world in which genetic modification, *ex situ* organ engineering, and cross-species transplantation were theoretical or experimental; in 2024, a patient received a genetically modified pig kidney under compassionate use in the United States [3], and European groups are preparing similar trajectories. The question facing the European Union is therefore not whether to update these instruments, but whether the update will keep pace with the biology or fossilise a step behind it. The answer will determine which patients gain access to emerging therapies, where that innovation is developed, and whether Europe’s regulatory architecture remains fit for a field whose boundaries are actively dissolving.

## From static oversight to risk-adapted biotechnology governance

European biotechnology governance has historically been defined by the precautionary principle. While this approach has ensured high safety standards, it has also imposed rigidity and slowed the translation of innovation into practice. The current reform trajectory signals a shift in regulatory logic itself: from process-based oversight toward risk-adapted, product-oriented governance, where biological entities are assessed less by how they are made and more by what they do and how they are used (Table 1).

**TABLE 1:** Regulatory evolution in EU biotechnology and transplantation.

​	Previous framework	Proposed revision
Regulatory philosophy	- Precautionary, *ex ante* risk containment - Uniform, process-based oversight with high procedural burden	- Risk-adapted, proportional, lifecycle governance - Differentiated risk tiers, simplified pathways for low-risk procedures
Logic for classification	- Method-based classification (how it is made) - Rigid categories (GMO/non-GMO; organ vs. non-organ) - Segmented regimes (medicines, devices, transplantation)	- Function- and risk-based evaluation (what it does) - Functional, context-dependent classification - Increasing convergence across regulatory domains
Governance model	- Static, rule-based regulation - Sector-specific and fragmented oversight	- Adaptive, data-driven, continuously updated oversight - Enhanced coordination across regulatory bodies
Decision timeline	Pre-authorisation dominant, slow adaptation	Conditional approval with iterative reassessment
Organ regulation	Linear pathway: donation → retrieval → storage → transplantation	Expanded continuum including *ex situ* organ processing and optimisation
*Ex situ* interventions	Not explicitly integrated	Explicitly included within regulatory scope

The move toward differentiated risk categories for genetically modified micro-organisms reflects this epistemic transition. Rather than treating genetic modification as a monolithic risk class, the emerging framework acknowledges heterogeneity in design, exposure, and potential clinical or environmental impact. This approach aligns with broader international trends, including European Medicines Agency (EMA) frameworks for gene therapy, which increasingly emphasise risk proportionality and context-specific evaluation [4].

Yet this shift introduces new tensions. Concepts such as “low-risk GMMs” or “qualified safety assumptions” create interpretative space that may lead to divergence across Member States. What appears as simplification at the EU level may translate into heterogeneity at implementation level. The core challenge therefore extends beyond scientific classification to maintaining coherence in governance across jurisdictions.

## Transplantation redefined: from retrieval to *ex situ* organ engineering

If upstream biotechnology is undergoing regulatory recalibration, transplant medicine is undergoing an even deeper ontological shift. Organ transplantation is no longer confined to retrieval, preservation, and implantation within narrow ischemic limits. Advances in machine perfusion, *ex situ* repair, and functional assessment are transforming the graft into a dynamic biological system-one that can be stabilised, evaluated, and even modified outside the body [5]. Evidence from liver transplantation shows that these technologies can increase organ utilisation and improve outcomes in selected settings [6–8]. More importantly, the imminent horizon holds the potential for interventions giving rise to a fully-fledged discipline of *ex vivo* organ medicine. In such a context both the time window and the conceptual scope of transplantation, will shift it from a discrete surgical act to a continuous process of biological intervention.

The proposed EU framework acknowledges this evolution by explicitly incorporating *ex situ* organ processing (Table 1), recognising that transplantation now spans a spectrum rather than a single event. Yet this expansion brings new regulatory challenges; the growing use of machine perfusion for optimisation or transformation before implantation must be anticipated.

Traditional governance was built around clearly defined stages—donation, allocation, retrieval, implantation. *Ex situ* intervention blurs boundaries, redistributing responsibility across device manufacturers, tissue establishments, clinical teams, and potentially pharmaceutical developers. Classification, in this context, becomes a determinant not just of compliance, but of whether innovation is feasible.

Formalising authorisation pathways for organ processing by competent authorities offers a pragmatic alternative to default classification as advanced therapy medicinal products, which would impose disproportionate burdens. But proportionality is key: overly complex procedures risk creating bottlenecks, delaying access, and ultimately disadvantaging patients.

A risk-adapted approach—focusing scrutiny -where uncertainty or risk is greatest is essential. At the same time, regulatory simplicity must be preserved for standard graft preservation. Established techniques, even when delivered through machine perfusion, should not face additional constraints that disrupt workflows without clear benefit.

By clarifying roles across transplantation centres, regulators, and other stakeholders, the directive can strengthen governance in an increasingly complex field. The introduction of clinical outcome monitoring in areas of residual uncertainty embeds a more adaptive, evidence-generating model-well suited to a domain defined by small patient populations and rapidly evolving technologies.

## Convergence of regulatory domains and fragmented biological reality

The strain that *ex situ* engineering places on transplantation governance is not an isolated problem; it is the first visible symptom of a broader structural tension. Regulating increasingly integrated biomedical systems through historically separate legal categories will inevitably strain the framework. Organs may now be perfused using devices, modified with biologics, assessed via digital biomarkers, and even improved through gene-based interventions. Each falling under different regulatory regimes with distinct evidentiary standards and timelines.

While the directive attempts to define boundaries, biology itself does not adhere to them. The result is growing regulatory ambiguity, particularly in advanced therapies where classification may depend more on framing rather than intrinsic biological differences. Increasingly, regulatory categories do not just evaluate innovation—they shape what innovation is possible.

## Evidence maturation drift: a hidden systemic risk

Beyond structural complexity lies a more subtle systemic risk: misalignment between regulatory acceleration and the maturation of clinical evidence. As adaptive and conditional approval pathways expand, market access may precede robust demonstration of clinical benefit.

This “evidence maturation drift” has clear precedents across biomedical fields, where early approval based on surrogate endpoints or technical validity has not consistently translated into meaningful improvements in patient outcomes. The issue is further exacerbated by the fundamentally different regulatory frameworks governing medical devices and pharmaceuticals. Unlike pharmaceuticals, which generally require extensive clinical evidence prior to market authorization, medical devices often enter the market through pathways that emphasize technical performance and substantial equivalence rather than robust demonstration of long-term clinical benefit. Consequently, disparities in evidentiary standards and regulatory oversight may contribute to the premature adoption of technologies whose real-world therapeutic value remains insufficiently validated.

Machine perfusion and organ modification strategies exemplify this tension: they are biologically plausible and technologically sophisticated, yet their long-term comparative effectiveness remains incompletely characterized. Conversely, excessive regulatory pressure may hinder the timely adoption of innovations with the potential to substantially improve clinical outcomes.

Maintaining an appropriate balance between rigorous regulation and biotechnological innovation therefore justifies robust post-authorisation evidence requirements. However, preventing regulatory drift requires more than post-market surveillance alone. It also demands a clear definition of the relevant clinical context, comparative evaluation whenever feasible, and mechanisms for continuous iterative reassessment.

The revised EU framework moves in this direction through strengthened post-authorisation evidence generation, including mandatory national transplant registries and structured data collection on donors, recipients, procedures, and long-term outcomes. Facilitated international data sharing under GDPR further supports this effort. In parallel, ESOT’s European transplant registry platform^1^ provides a foundation for harmonised, cross-border outcome analysis and continuous evidence generation. Overall, the critical role of scientific societies fostering prospective multicentric networks align with this need for evidence corroboration.

## Xenotransplantation: an emerging frontier without a dedicated framework

Perhaps the clearest stress test for the evolving European framework is xenotransplantation. The transplantation of genetically modified animal organs into humans is no longer theoretical but entering early clinical reality [3]. While feasibility is increasing, major challenges remain, particularly regarding immunological compatibility (impacting on long-term graft function), and infectious risk [9–11].

Regulatory approaches elsewhere, such as those of the U.S. FDA, emphasise lifelong surveillance and stringent zoonotic risk control [10, 11]. In Europe, xenotransplantation has no dedicated legal framework and is only indirectly governed through overlapping regimes on genetic modification, medicinal products, and organ transplantation. This results in a fragmented regulatory landscape, with oversight dispersed across instruments not designed for solid organ xenografts. At EU level, the Organ Transplantation Directive applies only to human organs, while other rules—such as those on xenogeneic cell therapies or medical devices incorporating non-viable animal tissues (Regulation (EU) 2017/745)—do not extend to xenotransplantation. Efforts to classify engineered animal organs as advanced therapy medicinal products remain conceptually unconvincing. Overall, this gap reflects longstanding European caution since the 1990s moratorium and generates significant legal uncertainty.

Furthermore, divergence exists across Member States. Netherlands maintains a *de facto* moratorium through its Special Medical Procedures Act [12], whereas France allows xenotransplantation under strict conditions within authorised research protocols. Established under Law No. 98–535 and Article L. 1127–2 of the Public Health Code, this framework ensures authorisation, traceability, and long-term monitoring, but remains strictly national and does not replace the absence of EU-level harmonisation.

This absence of explicit governance is not merely a technical gap but a structural weakness, as xenotransplantation raises specific ethical and public health risks—particularly cross-species pathogen transmission and the need for long-term recipient surveillance—that cannot be adequately addressed through fragmented regulatory oversight.

## Regenerative medicine and the shifting boundaries of organ definition

If xenotransplantation is the stress test the framework faces today, regenerative medicine is the one it will face next. As increasingly complex and functional constructs emerge, regulatory frameworks will need to move beyond traditional definitions based on anatomy alone, and instead account for function, origin, and intended use [13].

Advances in organ engineering and developmental biology further challenge regulatory categorisation. Organoids, chimeric systems, and stem-cell-derived tissues blur the boundary between repair and replacement, raising fundamental questions about what constitutes an “organ”.

This raises questions about whether regulatory frameworks should be anchored in anatomical structure, functional capacity, or developmental origin. As organ engineering progresses, particularly in vascularised and functional constructs, regulatory systems will increasingly need to accommodate entities that do not fit conventional definitions [13].

## Towards adaptive but accountable governance

The ambition of the proposed European framework is clear: to move from static precaution toward adaptive governance capable of keeping pace with biomedical innovation. This reflects a broader recognition that uncertainty is inherent to the life sciences.

The ambition of the proposed European framework is clear: to move from static precautionary regulation toward adaptive governance capable of keeping pace with biomedical innovation. This shift reflects a broader recognition that uncertainty is not an anomaly in life sciences, but a structural condition.

However, adaptability must not come at the expense of evidentiary discipline. The goal is not to trade speed for safety, but to balance them dynamically, to reach this equilibrium scientific and clinical expertise must remain central to decision-making, and regulatory decision-making and real-world clinical data must continuously inform regulatory assessment. Just as importantly, regulatory systems must remain open to revising earlier assumptions as knowledge evolves.

## Conclusion

Europe’s evolving regulatory architecture for biotechnology and transplantation marks a significant shift-seeking to align governance with the complexity of modern biology a domain where innovation sits at the convergence of genetic engineering, organ manipulation, and regenerative science.

Its success, however, will depend less on legislative intent than on implementation. Without robust mechanisms to align evidence, manage cross-domain convergence, and address emerging fields such as *ex situ* organ manipulation and xenotransplantation, there is a risk that regulatory acceleration will outpace understanding.

Three commitments would turn legislative intent into operational reality:

1. *Ex situ* organ processing should be governed by dedicated competent-authority authorisation pathways rather than by default classification as ATMPs; ATMP designation should apply only to genuinely novel interventions where the risk profile warrants it, so that established machine perfusion and related optimisation techniques are not burdened with disproportionate procedural requirements. Importantly, current EMA guidance for cells and tissues (EMA/681445/2011) already states that products which do not undergo substantial manipulation and are re-administered to fulfil the same essential function will generally not be considered ATMPs. In this regard, machine perfusion procedures clearly fulfil these criteria and should therefore not automatically fall within the ATMP regulatory framework.
2. Xenotransplantation requires an integrated European framework that explicitly bridges GMO, ATMP, and organ regimes, with mandatory cross-border recipient registries, lifetime zoonotic surveillance, and harmonised consent and containment standards — rather than the current dispersal of oversight across instruments not designed for it.
3. Adaptive authorisation must be paired with mandatory post-authorisation evidence infrastructure: national and European registries, structured long-term outcome collection, and pre-specified reassessment thresholds at which conditional approvals are either confirmed, narrowed, or withdrawn. Without this paired commitment, flexibility becomes drift.

The real challenge is not whether Europe can modernise its framework — but whether it can build one that is as adaptive, integrated, and self-correcting as the biology it aims to govern.

## Footnotes

1
https://esot.org/esot-registry/
